# The Road to Dissemination: The Concept of Oligometastases and the Barriers for Widespread Disease

**DOI:** 10.3390/cancers14082046

**Published:** 2022-04-18

**Authors:** Hamza AlGhamdi, Jennifer Dhont, Mohammad Krayem, Pauline De Bruyn, Benedikt Engels, Dirk Van Gestel, Robbe Van den Begin

**Affiliations:** 1 Radiotherapy Department, Jules Bordet Institute, Université Libre de Bruxelles, 1070 Brussels, Belgium; hamza.alghamdi@bordet.be (H.A.); pauline.debruyn@bordet.be (P.D.B.); dirk.vangestel@bordet.be (D.V.G.); 2 Oncology Center, King Faisal Medical City, Abha 62523, Saudi Arabia; 3 Medical Physics Department, Jules Bordet Institute, Université Libre de Bruxelles, 1070 Brussels, Belgium; jennifer.dhont@bordet.be; 4 Laboratory of Clinical and Experimental Oncology (LOCE), Jules Bordet Institute, Université Libre de Bruxelles, 1070 Brussels, Belgium; mohammad.krayem@bordet.be; 5 Radiotherapy Department, Universitair Ziekenhuis Brussel, Vrije Universiteit Brussel, 1050 Brussels, Belgium; benedikt.engels@gmail.com

**Keywords:** oligometastasis, metastasis, stereotactic radiotherapy, organ tropism, cancer biology

## Abstract

**Simple Summary:**

Oligometastatic disease is an intermediate state of metastatic dissemination with a limited number of metastatic sites and extent of disease. Tumor cells need multiple capabilities in order to migrate, survive and evolve to macroscopic metastases. These capabilities are acquired by evolutionary mechanisms and are associated with several clinical factors and biomarkers. Better understanding of these properties and biomarkers may help to select patients that can benefit from local ablative therapies, which have shown to be a promising approach in recent clinical evidence.

**Abstract:**

Over the last years, the oligometastatic disease state has gained more and more interest, and randomized trials are now suggesting an added value of stereotactic radiotherapy on all macroscopic disease in oligometastatic patients; but what barriers could impede widespread disease in some patients? In this review, we first discuss the concept of oligometastatic disease and some examples of clinical evidence. We then explore the route to dissemination: the hurdles a tumoral clone has to overtake before it can produce efficient and widespread dissemination. The spectrum theory argues that the range of metastatic patterns encountered in the clinic is the consequence of gradually obtained metastatic abilities of the tumor cells. Tumor clones can obtain these capabilities by Darwinian evolution, hence early in their genetic progression tumors might produce only a limited number of metastases. We illustrate selective dissemination by discussing organ tropism, the preference of different cancer (sub)types to metastasize to certain organs. Finally we discuss biomarkers that may help to distinguish the oligometastatic state.

## 1. Introduction: The Spectrum Theory and the Rationale of Oligometastatic Disease

Metastatic dissemination is the development of secondary cancer lesions at a distance from the primary neoplasm. This process is believed to be the most common source of failure in the treatment of cancer and the principal cause of cancer mortality [1]. During the last century, our paradigm of the metastatic process has evolved. The spectrum theory, proposed by Hellman in 1994, synthesizes multiple former theories [2]. It suggests that cancers range between a local disease and a disease that is already systemic at the moment of diagnosis. The metastatic capabilities of the tumor depend on its growth and progression, during which it gradually acquires the properties needed for efficient and widespread dissemination. The evolution of these abilities [3] would not only take place in the preclinical stage, but could also continue during the period in which the disease is clinically overt. Lymph node involvement is often a prognostic parameter not only because it indicates more aggressive tumor biology, but also because persistent lymph node disease can be the source of distant metastases. This explains the benefit of regional therapies in certain tumors.

**Figure 1:**
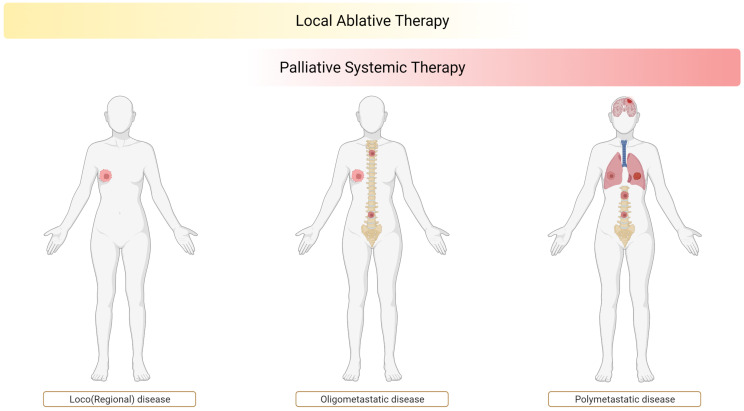
Oligometastatic state as an intermediate state between localized disease and widespread dissemination.

As a logical consequence of the spectrum theory and the multistep nature of cancer progression, the oligometastatic state was proposed, being an intermediate between localized disease and widespread dissemination [2] as illustrated in Figure 1. If the traits needed for efficient dissemination can develop gradually, early in the genetic progression tumors might produce metastases limited in number. Additionally, only specific organs may allow colonization of these early metastases, in accordance with the “seed and soil” principle [2]. Limitation of the metastatic spread may therefore not only happen because of anatomical reasons (e.g., limitation to liver metastases in a case of colorectal cancer) but also for biological reasons.

The clinical implication of the oligometastasis hypothesis is that local treatment such as surgery, cranial stereotactic radiosurgery (SRS), extracranial stereotactic body radiotherapy (SBRT), or other ablative approaches at the metastatic level, may improve the prognosis of patients with oligometastatic disease. Several surgical series showed extensive survival following resection of secondary lesions like liver metastases from colon primaries [4] or secondary lung lesions [5]. However, no survival benefit was achieved in the PulMiCC trial, a randomized study on lung lesion metastasectomy [6].

SBRT and SRS are non-invasive and highly focused treatments [7]. Long survival was also reported after stereotactic radiotherapy for selected patients with oligometastases [8]. Recently, several randomized phase II trials confirmed that irradiating all visible oligometastases may provide a clinical benefit. For example, the SABR-COMET trial [9] randomized 99 patients with a controlled primary tumor and ≤5 metastasis between standard of care alone (SOC) versus standard of care plus SBRT to all metastatic sites. After a median follow-up of 51 months, the 5-year overall survival rate was 17.7% in the SOC arm versus 42.3% in the SBRT arm. ORIOLE, another example of a phase II trial [10], has randomized 54 men with PET PSMA confirmed oligometastatic prostate cancer (≤3 metastases) to receive either SBRT or observation. Progression at 6 months occurred in 7 of 36 patients (19%) in the SBRT group, versus 11 out of 18 patients (61%) in the observation group (*p* = 0.005). Treatment with SBRT also improved median progression-free survival (not reached vs. 5.8 months). Several randomized phase III trials are currently ongoing to further evaluate the benefit of adding focal radiotherapy to the standard treatment in oligometastatic disease of various primary tumors.

Evidence of the existence of an oligometastatic state and the benefit of local therapy is thus emerging. To better understand how an oligometastatic state can exist, we discuss the metastatic process in general: which barriers exist that can make a difference between limited and widespread dissemination, and how cancer cells acquire the capacities to overcome these hurdles. We take a look at organ tropism, an example of specific dissemination, and discuss the developing biomarkers that may play a role in oligometastatic disease.

## 2. The Metastatic Cascade

**Figure 2:**
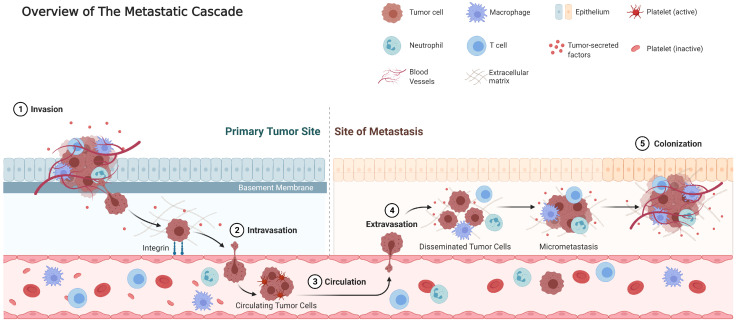
Overview of the steps in cancer metastasis. (Adapted from Fares et al. [11]).

To better understand the possibility of limited metastatic dissemination, we need to comprehend the metastatic development process, the so-called metastatic cascade. For cancer cells to metastasize, they have to be able to survive on the primary site, then acquire the ability to detach and to move into the blood or lymphatic stream where they have to sustain the dynamic fluid conditions and evade the immune system to reach the secondary site. Finally, they have to escape the bloodstream to re-implant and survive in a secondary site [11], as illustrated in Figure 2.

Although this sequence appears chronological from a biological viewpoint, the required tumoral traits for this process are not necessarily acquired in this order [12]. Several abilities overlap with those needed for local invasion, but as illustrated by the genome analysis of pancreatic metastases, seeding of metastases probably necessitates additional mutations on top of these required to develop a primary tumor [13].

A first important trait is diminished cellular adhesion, exhibited by most carcinoma cells, which permits a cancer cell to move more freely. Loss of E-cadherin-mediated adhesions and modification of integrin activity are examples of the mechanisms tumors use to acquire mobility, which is an essential part of the metastatic process [14]. Uppal et al. studied clinical oligometastasis samples and identified regions in the 14q32 microRNA cluster as co-regulators of multiple metastatic pathways. The oligometastatic phenotype was indeed associated with suppression of cellular adhesion, invasion, and motility pathways [15].

An acquired freedom can be exploited when the cell obtains an increased motility. Cytoskeletal changes, but also localized proteolysis play a role in this process, e.g., by secretion of extracellular matrix metalloproteinases (MMPs) and cathepsins [16]. Proteolysis is very useful to disrupt the extracellular matrix and the basement membrane, which clears the way for local invasion. In addition, immunosuppressive cytokines are produced to evade immune cells and inflammation pathways are activated which promote metastasis [17]. Overall, inflammation promotes multiple principal hallmarks of cancer [3].

To reach a distant site and induce a metastasis, malignant cells must infiltrate vessels. This is eased by the creation of new blood and lymphatic vessels by the tumor. Angiogenesis is needed for a tumor to grow beyond the size of a few millimeters, since otherwise it is limited to the diffusion limits of oxygen and nutrients. Lymphangiogenesis can also take place, providing a tortuous network of vessels that drains interstitial fluids to lymph nodes and finally to the blood circulation, hence providing another metastatic route [18]. The metastatic infiltration of lymph nodes is an adverse prognostic factor as it indicates an aggressive disease. Nevertheless, it is currently assumed that direct hematogenous dissemination is the principal metastatic route for distant metastases. Hypoxia and nutrient deprivation in the tumoral, endothelial, and stromal cells trigger an “angiogenic switch”. Outgrowth of new blood vessels from nearby vessels is elicited by release of angiogenic factors, such as fibroblast growth factor (FGF), vascular endothelial growth factor (VEGF), platelet derived growth factor (PDGF), stromal derived factor 1 (SDF-1), and angiopoietins [19]. The excessive production of vasogenic factors results in a chaotic, tortuous network of immature vessels [20]. The conditions of aberrant endothelial organization and structure, deficient pericytes, and defective basement membranes culminate in leaks, fluid extravasation, and platelet activation.

Once tumor cells have migrated to nearby vessels and invaded the circulatory system, they become circulating tumor cells (CTC) and have to survive the transit to distant sites. They may do this as single cells or in tumor cell clusters [16]. Main hazards are oxidative stress, physical damage from shear stress, and attack by the immune system. Cancer cells modify their metabolism to counter the oxidative stress [16]. The evasion of the latter two threats may be facilitated by the co-optation of blood platelets, using them as shields. Platelets accumulate on embolic cancer cells, protecting them from clearance by the immune system, and increase entrapment in distant tissues. They may also assist in the adhesion of circulating tumor cells to vascular endothelium, enabling extravasation. Activated platelets indeed connect to tumor cells and endothelial cells by integrins, and secrete factors that stimulate extravasation [21]. These mechanisms illustrate the two potential pathways for a metastatic cell to invade the surrounding tissue, once arrived at a distant site. One pathway is the homing of malignant cells, mediated by chemokines and resulting in the adhesion of malignant cells to the endothelium via surface receptors. Subsequent extravasation is aided by increased vascular permeability, mediated by VEGF, COX2, and other molecules. This homing process might be partially responsible for the propensity that different primary tumors have for metastasization to certain target organs [18]. The main pathway, however, is considered to consist of a crude mechanical entrapment of tumor cells in capillaries of distant organs, with or without associated platelets. A lesion may then grow intravascularly until the tumor bursts through the vessel wall [12]. CTCs originating in the gut encounter a first capillary bed in the liver, for most other organs this is the lung. This concept of capillary entrapment contributes to the high frequency of metastases in liver and lung.

An abundant number of CTCs may be spreading from the primary tumor, but only a small fraction will infiltrate distant tissues and survive, the so-called disseminated tumor cells (DTCs). In addition, even when multiple DTCs are present at diagnosis, only some will become overt metastases [18].

Once present in a niche, metastatic cells would not survive or grow without stimulation of certain pathways. To achieve this, cells may produce autocrine pathway activators themselves or recruit stromal cells to do so. Physical contacts with stromal cells may provide support as well. Other mechanisms are the enhancement of pro-metastatic pathways by epigenetic alterations or by expression of microRNAs [16]. Not infrequently, metastases appear only years after resection of the primary tumor. This demonstrates that DTCs can stay in a “dormant state” for very long periods. Several explanations were put forward: an inadequate local environment (for example lack of above-mentioned growth factors or cell contacts), insufficient neovasculature, or immune system surveillance that keeps tumors at a microscopic size. Analysis of matched primary and metastatic breast cancer samples showed that (successful) metastases had a lower immune score and increased immune-permissive cells [22]. Two forms of dormancy are thought to exist: “Cellular dormancy” consists of non-proliferating isolated DTCs, as found in the bone marrow in several carcinoma types. Such solitary disseminated cells seem indeed capable of entering a cell-cycle arrest [12]. This appears to result from an incompatibility with the local “soil,” since these dormant cells can form tumors when re-implanted in the primary tumor tissue [23]. “Tumor mass dormancy” on the other hand, would involve micrometastases without enlargement, due to insufficient vascularization or to proliferation being compensated by apoptosis or immune defenses. Although some stromal signals were uncovered that influence the beginning, continuation, or termination of a dormant state, there is still a relative paucity of data about this process [16]. In particular, it is unknown how a continuously quiescent DTC population can suddenly evolve to macroscopic metastases.

The final step of the metastatic cascade is the overt colonization of a distant site by an infiltrating or previously dormant cell. Besides overcoming general issues like insufficient nutrients and immune defenses, metastatic cells may need organ-specific traits to flourish at a certain site. This contributes to the specific metastatic patterns exhibited by different tumor types, as discussed below under “Organ tropism”. Several studies used full genome analysis of different metastases within the same patient to examine their relationship, specifically how they descend from each other. This analysis showed family trees with organ-specific branches, in other words, genetic changes might determine to which organs metastasization occurs [13,24]. Finally, similar survival principles apply for a growing metastatic lesion as for the primary tumor. The tumor-stroma interactions equally play a central role, allowing the metastases to rally the microenvironment to their cause [25].

## 3. The Evolution to Metastatic Capabilities

As described above, cancer cells several traits to metastasize successfully, abilities that either increase the cells potential, or that influence other cells to collaborate. The acquisition of these traits is driven by genetic and epigenetic evolution, of which the principles are comparable with the Darwinian evolution of species [26]. Two principles form the base of this type of evolution.

**Figure 3:**
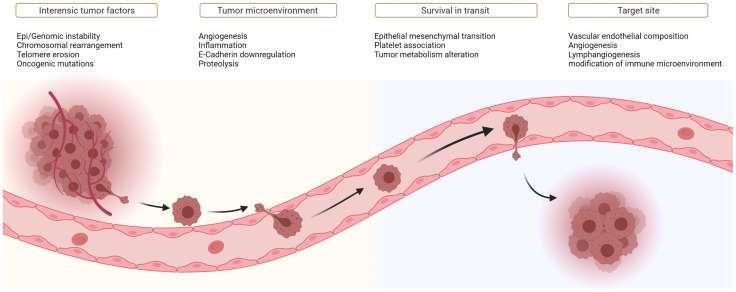
Factors that influence metastatic capability.

The first is the presence of a genetic variety. In malignant progression, this diversity is abundantly present because of the genetic instability of the genome, which constitutes one of the “enabling hallmarks” of cancer [3] and results in many different clonal lines. Evidence is indeed found that genetic heterogeneity exists among metastasis-initiating cells [13], within individual metastases and between different metastatic sites within the same patient [27,28]. Frequent changes are epigenetic alterations, chromosomal rearrangements [3], telomere erosions, and mutations, deletions, or amplifications in different genes [22]: tumor-suppressor and DNA-repair genes, and genes responsible for tumor metabolism (aerobic glycolysis), causing toxicity for surrounding normal cells [29] (Figure 3). However, the research on specific metastasis-driving genes is still ongoing. Mutations in TP53 are connected to the metastatic process in prostate cancer and in some colorectal cancers, possibly because TP53 dysfunction is associated with chromosomal instability and chromothripsis [30]. Likewise, It is possible that epigenetic alterations and other modifications in gene expression may be the dominant source of selectable pro-metastatic traits during clonal evolution [12,31].

Stochastically, an (epi)genetic change leading to a certain trait will arise sooner or later in one or several clonal branches. However, of the millions of malignant cells in a tumor, only a minor part may obtain all the necessary traits for metastatic development [32].

The second principle of cancer evolution is “survival of the fittest”, selection of clonal lines with genetic perks. These may constitute a survival benefit (as in the Darwinian “natural selection”) or a proliferative advantage (which may be compared to “sexual selection”). In the somatic evolution of cancer cells, the selection is imposed by cell-intrinsic and/or extrinsic pressures. Examples of intrinsic obstacles are genotoxic stress induced by oncogenes, growth inhibitory, apoptotic and senescence pathways, and telomere attrition [12]. Evasion of these tumor-suppressive pathways is one of the hallmarks of tumors [33]. Extrinsic barriers hinder the development of tumors at the primary site, but mechanisms that bypass these barriers may also facilitate development in distant sites [12]. These challenges may be chemical (hypoxia, low pH, free radicals), physical (basement membrane, interstitial pressure, tensional forces), or biological (immune system, cytokines) (Figure 2). One example is the generation of reactive species of nitrogen and oxygen, both by infiltrating inflammatory cells and rapidly proliferating tumor cells. Hypoxia is a strong selective pressure in tumors, promoting the outgrowth of malignant cells with increased resistance to apoptosis. For example, hypoxia induces stabilization of a hypoxia inducible factor-1 (HIF-1) transcriptional complex. This complex stimulates multiple steps in the metastatic cascade, e.g., anaerobic metabolism, angiogenesis, cell survival, and invasion [34,35].

Cancer treatments may impose an extrinsic selection pressure as well, possibly resulting in the emergence of resistant clones [36], which may further progress and spread to other lesions [16,37,38]. If the progression is limited, the term “oligoprogression” is used. Oligoprogression is a situation in which a patient had either widespread or genuine oligometastatic disease, for which systemic therapy was able to control a part of the metastases, but a limited number does progress at a certain point during therapy [39]. Local treatment of these resistant lesions may therefore allow to extend the benefit the ongoing systemic treatment, to which the majority of the disease is still sensitive. This approach is becoming more widely used, especially in non-small cell lung cancer and renal cell cancer, and a number of trials are ongoing to confirm the benefit for the patient [40].

Two models exist for the phylogenetic relationship between a primary tumor and a derived metastasis: the linear progression model and the parallel progression model. In the linear progression model, the clone with tumor-initiating capacity develops relatively late in the tumor development. The genetic differences (divergence) between the primary and secondary lesion will therefore be small. At the other end of the spectrum, in the parallel progression model, a clonal line seeds out earlier during tumor progression. Afterwards, genetic evolution in the metastatic lesion continues in parallel with the evolution of the primary cell line, resulting in larger genetic divergence [30]. These models are the two extremes of a spectrum, and an intermediate pattern may equally take place. The testing of these models is, however, limited due to heterogeneity in the primary tumor. Genomic heterogeneity within tumoral lesions can be very extensive and complicates the characterization of its properties [28]. When genetic testing is done on a single sample of the primary tumor, this may coincidentally be a sample of a different clonal line than the one that produced the metastatic clone [41]. A large genetic divergence between a metastatic cell and a primary cell may thus be proof of parallel genetic progression or due to limitations in sampling [30].

In conclusion, the journey to successful metastases is fueled by the principles also found in the evolution of species. Figure 3 shows an overview of several factors associated with the development of metastatic capabilities of tumors along their dissemination pathway.

## 4. Organ Tropism: An Example of Selective Metastases

The concept of limited metastatic potential in oligometastatic disease is illustrated by a comparable, well-documented phenomenon of organ-selective spread. As mentioned above, some primary tumors have a propensity to metastasize—at least in a certain part of the disease history—to certain organs solely. This principle was already clear in the 19th century [42], and has remained a research area of interest called “organ tropism” [43].

As example, prostate cancer has a striking tendency to metastasize to bone, sarcomas to the lungs and uveal melanoma to the liver. Melanomas, lung and breast adenocarcinomas on the other hand, tend to seed to multiple organs [44]. Differences in metastatic kinetics can also be noticed. For example, brain and other metastases often appear early in the history of lung cancer, while brain lesions typically occur only late in metastatic breast cancer [16].

Extrinsic mechanisms, such as anatomical properties, have their part in organ tropism. As mentioned, liver and lung contain the first capillary beds for specific parts of the circulation and may filter a disproportionately large share of tumoral embolisms from different organs. The composition of the vascular wall also varies per organ. The capillaries in liver and bone marrow are more porous for instance, while the endothelium of lung capillaries have tight junctions and a basement membrane. In the brain, pericytes and astrocytes provide additional support and together form the blood-brain-barrier. Nevertheless, there is a clear discrepancy between vascular anatomy and organ susceptibility for metastases [42]. This formed the base for the seed-and-soil hypothesis. By illustration: kidneys, liver and brain equally receive approximately 10–20% of blood flow, but their metastatic patterns are very different [45]. The most important factors in organ tropism are therefore intrinsic to the tumor and its metastatic capabilities [32,45]. In clinical practice, an additional demonstration is given by the organ specificity of various tumor subtypes. In lung cancer, adenocarcinoma seeds the brain and adrenal gland more often than squamous carcinoma does. Between the different subtypes of breast cancer, luminal A, and luminal B tumors have a higher tendency to form bone metastases, and HER2+ breast cancer induces a higher frequency of liver metastases [45]. In addition, certain oncogenic mutations seem to influence organ tropism [16]. In pancreatic cancer, phylogenetic trees of the metastatic origin show organ-specific branches [13].

Several studies in mouse models also show the importance of the intrinsic metastatic capabilities for organ specificity [45]. For example, KRAS-mutant colon cancer has a propensity to colonize the lungs from existing liver metastases [46]. Intrinsic abilities allow CTCs to cross physical barriers, survive at distant sites, interact successfully with organ-specific cells, and eventually colonize the distant organ. According to Gupta, there are two important mechanisms for selective dissemination [12]. Firstly, the productive interaction with host tissue to extract growth and survival advantages. Various examples can be found in a paper by Lambert et al. [47]. Secondly, the creation of pre-metastatic niches is also organ-specific [48]. For example, integrins can target exosomes to specific organs to unload their cargo, preparing a pre-metastatic niche to host tumor cells [16]. The combination of organ-specificity of the niches and the metastases determines the total dissemination picture [48]. Nevertheless, the overwhelming majority of disseminated tumor cells will never achieve colonization, since the rare surviving cells will arrive at soil that is tolerant at best [45].

Organ tropism is therefore a well-documented concept, based on the same foundations as oligometastatic disease: only a small proportion of circulating tumor cells has the intrinsic capacity to succeed in infiltrating, surviving, and eventually overtaking a distant organ [45]. One may imagine that both oligometastases and organ-confined dissemination are disease states side by side on the spectral scale that ranges from local disease to overt multi-organic dissemination.

## 5. Biomarkers in Oligometastatic Disease

Key questions remain in our understanding of how best to identify patients with the oligometastatic disease who will benefit from oligometastatic treatment.

Since no validated biological biomarker for the identification of patients with true oligometastatic disease is clinically available, the diagnosis of oligometastatic disease is currently based solely on imaging findings. Imaging studies are optimized to comprehensively assess metastatic sites, disease burden, and response to neoadjuvant treatment in oligometastatic disease setting, features that can be considered as the first available biomarkers [49]. However, the range of the metastatic spectrum qualifying as oligometastatic disease is not yet clearly defined. Usually only the number of hematogenous metastases is considered, with most studies including patients with maximum 3 or maximum 5 lesions. Whether and how other factors such as the number of involved organs and the speed of progression should be incorporated is not yet clear [50,51]. In addition, identifying other biomarkers to distinguish truly oligometastatic patients from patients with occult disseminated disease would be of great interest.

[^18^F]FMCH PET/CT radiomic analyses provided information about tumor heterogeneity of prostate cancer (PCa) recurrence, entailing discriminant ability in differentiating the disease according to the site of recurrence and the tumor burden [52]. The study suggests that the definition of oligometastatic PCa should include patients with no more than three lesions. Indeed, oligometastatic patients defined as having up to five lesions, exhibited a heterogeneity comparable to plurimetastic patients [52]. The two randomized landmark trials that showed a benefit of metastasis-directed therapy in PCa, used indeed an upper threshold of 3 metastases as inclusion criterion [10,53]. Some predictors have been identified on the molecular level. In the case of liver metastasis from colorectal cancer, KRAS, and BRAF mutations have shown to be associated with accelerated metastatic progression and poorer survival [54,55]. In another study by Pitroda et al. [56], three subtypes of colorectal cancer liver metastases were identified using an integrative molecular analysis. Patients with metastases that have signs of immune activation presented the best overall survival, whereas patients with tumors that demonstrated VEGFA amplification or NOTCH1 and PIK3C2B mutations with E2F/MYC activation had a worse prognosis. These subtypes were identified with integrated transcriptional analysis of mRNA and microRNA networks and can complement clinical risk stratification to predict survival after liver metastasectomy [56]. Lussier et al. identified microRNA profiles in patients with various cancer types, predicting an oligo- versus polymetastatic progression, and more particularly the rate of metastatic progression [57,58]. Dhondt and colleagues [59] described a microRNA signature to identify oligometastatic prostate cancer, while Uppal et al. identified four microRNAs encoded in the 14q32 locus that are associated with an oligometastatic phenotype in clinical metastasis samples [15].

Even more interesting would be the ability to distinguish a oligometastatic state through liquid biopsies: detecting relevant biological molecules and macrostructures in peripheral blood, such as circulating tumor DNA (ctDNA), circulating microRNA, circulating free RNA, extracellular vesicles, or circulating tumor cells [60]. Recent studies have indicated the clinical utility of ctDNA for molecular residual disease assessment, monitoring recurrence, and treatment response in patients, with emerging applications in oligometastatic patients [61,62,63,64,65]. The remarkable advances in ctDNA-derived oncogenomic profiling technology over the past years improved its specificity, target quantification and cost-effectiveness [62]. In addition, liquid biopsy can be a diagnostic tool in oligometastatic patients whose limited metastases are difficult to biopsy. This can serve for example in non-small cell lung cancer to seek oncogene mutations that have an impact on therapy [60]. The most tangible application of ctDNA in oligometastatic disease is the detection of minimal residual disease after metastasis-directed therapy. This detection could predict the utility of adjuvant systemic therapy and therefore be used to personalize therapy [66]. Afterwards, ctDNA could serve to detect recurrence ahead of radiological progression [67].

Finally, blood chemistry tests could provide prognostic value. Serum lactate dehydrogenase was analyzed by Nieder and colleagues in patients with oligometastatic brain metastases [68] and found to be associated with survival, because this biomarker may reflect the total burden of malignant disease. The same group validated a LabBM score (serum lactate dehydrogenase, C-reactive protein, albumin, hemoglobin, platelets) in patients with a limited number of brain metastases [69]. In another study of 403 oligometastatic patients that received SBRT, the prognostic value of the modified Glasgow Prognostic Score (mGPS) and the neutrophil–lymphocyte ratio (NLR) were investigated. mGPS is a risk score combining high CRP (>10 mg/L) and hypoalbuminemia (<35 g/L). Both scores correlated with overall survival (both *p* = 0.02), but did not yield additional prognostic value to a multivariate model of clinical parameters: histology, presence of brain metastases, Performance Score, gender, and timing of metastases (synchronous versus metachronous) [8].

## 6. Conclusions

The understanding of metastatic dissemination has increased since the inception of the oligometastatic principle in the nineties. Tumoral cells need multiple capabilities to migrate, survive, and form macroscopic metastases. These capabilities are acquired by evolutionary mechanisms. Due to tumor heterogeneity, the capacity to metastasize differs between patients and evolves with the disease course. Within this spectrum, patients with limited metastases, oligometastases, may profit from aggressive local therapies, which start to show great potential in well-selected populations. In the future, biomarkers may help to select patients for local metastasis-directed therapy.

## Abbreviations

The following abbreviations are used in this manuscript:

SRS	Stereotactic radiosurgery
SBRT	Stereotactic body radiotherapy
SOC	Standard of care
MMPs	Matrix metalloproteinases
CTC	Circulating tumor cells
FGF	fibroblast growth factor
VEGF	vascular endothelial growth factor
PDFG	platelet derived growth factor
SDF-1	stromal derived factor 1
COX2	cyclooxygenase-2
DTCs	disseminated tumor cells
PDFG	platelet derived growth factor
HIF-1	hypoxia inducible233factor-1
PCa	prostate cancer
ctDNA	circulating tumor DNA
